# XENOBREAST Trial: A prospective study of xenografts establishment from surgical specimens of patients with triple negative or luminal b breast cancer

**DOI:** 10.12688/f1000research.26873.2

**Published:** 2021-03-10

**Authors:** Hugo Veyssière, Judith Passildas, Angeline Ginzac, Sejdi Lusho, Yannick Bidet, Ioana Molnar, Maureen Bernadach, Mathias Cavaille, Nina Radosevic-Robin, Xavier Durando

**Affiliations:** 1 Université Clermont Auvergne, INSERM UMR 1240 « Imagerie Moléculaire et Stratégies Théranostiques », Centre Jean Perrin, Clermont-Ferrand, 63011, France; 2 Division de Recherche Clinique, Délégation Recherche Clinique & Innovation, Centre Jean Perrin, Clermont-Ferrand, 63011, France; 3 Centre d’Investigation Clinique, UMR501, F-63001, Clermont-Ferrand, 63011, France; 4 Département d’oncogénétique, Laboratoire d'Oncologie Moléculaire, Centre Jean Perrin, Clermont-Ferrand, 63011, France; 5 Département d’Oncologie Médicale, Centre Jean Perrin, Clermont-Ferrand, 63011, France; 6 Département d’anatomie et de cytologie pathologiques, Centre Jean Perrin, Clermont-Ferrand, 63011, France

**Keywords:** Patient-Derived Xenografts, Triple negative breast cancer, Luminal B breast cancer, Interventional research

## Abstract

**Introduction: **Patient-derived xenografts (PDX) can be used to explore tumour pathophysiology and could be useful to better understand therapeutic response in breast cancer. PDX from mammary tumours are usually made from metastatic tumours. Thus, PDX from primary mammary tumours or after neoadjuvant treatment are still rare. This study aims to assess the feasibility to establish xenografts from tumour samples of patients with triple negative or luminal B breast cancer in neoadjuvant, adjuvant or metastatic setting.

**Methods: **XENOBREAST is a single-centre and prospective study. This feasibility pilot trial aims to produce xenografts from tumour samples of patients with triple negative or luminal B breast cancer. Patient enrolment is expected to take 3 years: 85 patients will be enrolled and followed for 28 months. Additional blood samples will be taken as part of the study. Surgical specimens from post-NAC surgery, primary surgery or surgical excision of the metastases will be collected to establish PDX. Histomolecular characteristics of the established PDX will be investigated and compared with the initial histomolecular profile of the collected tumours to ensure that they are well-established.

**Ethics and dissemination: **XENOBREAST belongs to category 2 interventional research on the human person. This study has been approved by the Sud Méditerranée IV – Montpellier ethics committee. It is conducted notably in accordance with the Declaration of Helsinki and General Data Protection Regulation (GDPR). Study data and findings will be published in peer-reviewed medical journals. We also plan to present the study and all data at national congresses and conferences.

**Registration:** ClinicalTrials.gov ID NCT04133077; registered on October 21, 2019.

## Introduction

Breast cancer can be classified into different molecular subtypes according to gene expression: luminal A (Hormone Receptor positive (HR+)/human epidermal growth factor receptor 2 negative (HER2-), with high HR expression and low levels of the protein Ki-67), luminal B (HR+ either HER2+ or HER2- with high levels of Ki-67 and with low HR expression), HER2-enriched (amplification of *ERBB2* gene, regardless of HR status) and triple-negative (HR-/HER2-). Luminal B and triple-negative tumours account respectively for approximately 20% and 10–15% of all breast cancers ^1– 3^. Although rarer than luminal A breast cancers, luminal B and triple-negative tumours are often high-grade tumours with a poorer prognosis ^1^.

The establishment of patient derived xenografts (PDX) could be useful to discover new treatments and strategies needed in the fight against these subtypes of breast cancer. PDX are derived from tumour tissue in which the tumour architecture and the proportion of cancer and stromal cells are both maintained: advantages not found in cell lines. Therefore, PDX effectively model intra- and inter-tumoural heterogeneity ^4, 5^.

Thus, PDX are used to answer questions such as the contribution of tumour heterogeneity to therapeutic response, patterns of tumour progression during metastatic progression and mechanisms of treatment resistance ^6, 7^.

Most of the available PDX, derived from breast cancer, were generated from metastatic tumours. In particular, it may allow the identification of eventual molecular therapeutic targets in metastatic setting.

It is also necessary to have PDX models from primary breast lesions that are resistant to neoadjuvant therapies. Recent prospective studies show that PDX can be obtained from neoadjuvant breast tumours and demonstrate the feasibility of tumour sequencing in these situations ^8, 9^. Breast cancer patients with residual disease after neoadjuvant chemotherapy (NAC) have an increased risk of recurrence. Similarly, high-grade breast tumours treated by primary surgery are very rare, poorly known, and aggressive.

The production of PDX from post-NAC residual breast tumours or from high-grade breast tumours will provide data on the molecular characteristics of these tumours with a high risk of recurrence.

In this study, we want to establish PDX from tumour samples of patients with triple-negative and luminal B breast cancers in neoadjuvant, adjuvant or metastatic settings. In addition, to verify whether or not the PDX obtained is consistent with the original tumour, we will study the tumour exomes of both the PDX and the original tumour. The study of the patients’ constitutional exome will serve as the basis for this comparison and is an essential element in the overall somatic analysis.

## Methods

### Study design

This is a single-centre prospective trial designed to establish xenografts from surgical specimens of patients with triple negative or luminal B breast cancer in neoadjuvant, adjuvant or metastatic setting. Patient enrolment is expected to take 3 years: 85 patients will be enrolled and followed during 28 months.

**Figure 1.**
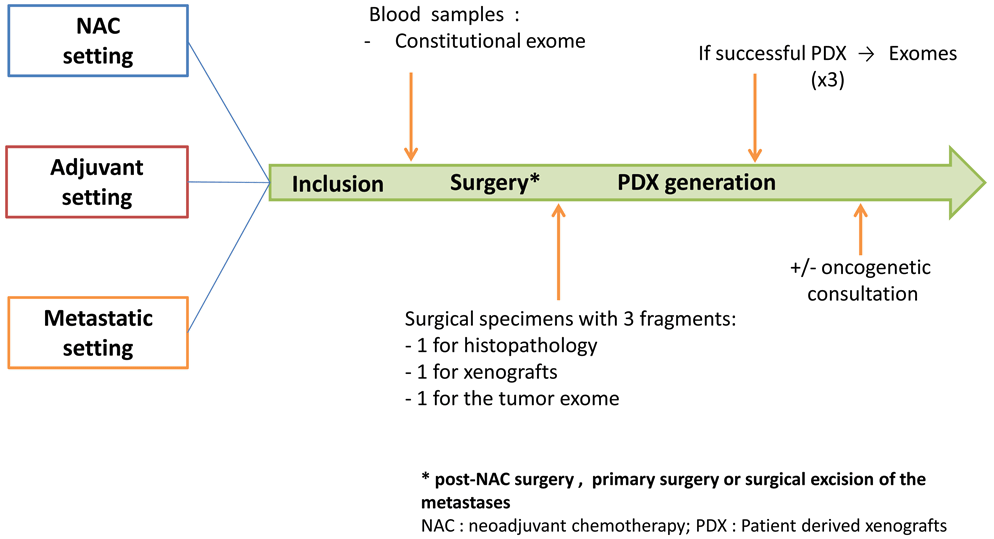
Design of the XENOBREAST study. Adult women with triple negative or luminal B breast cancer in neoadjuvant, adjuvant or metastatic setting will be included. In all the settings, patients will go through a pre-operative biological assessment. Blood samples will be used to perform sequencing of the patient's constitutional exome. At the end of the surgery (post-NAC surgery, primary surgery or surgical excision of the metastases), a sample of the surgical specimen will be taken to generate patient-derived xenografts (PDX) and another one to sequence the patient's tumour exome.

Study design is presented in Figure 1. The management of patients in the study may vary depending on the setting: neo-adjuvant, adjuvant or metastatic. In all the settings and as a part of their medical follow-up, patients will go through a pre-operative biological assessment. For patients in metastatic setting, this assessment will be performed before surgical excision of the metastases. Blood samples will be used to perform sequencing of the patient's constitutional exome. At the end of the surgery, one sample of the surgical specimen will be taken to generate PDX and another one to sequence the patient's tumour exome.

Participants can withdraw at any time. Data obtained will be retained with consent, and any reasons given for withdrawal will be recorded.

### PDX generation

**Figure 2.**
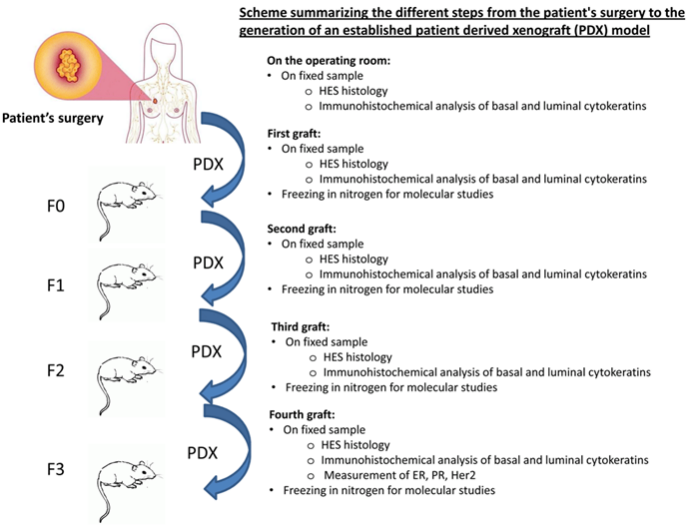
Scheme summarizing the different steps from the patient's surgery to the generation of an established patient-derived xenografts (PDX) model. Fresh surgical tissue will be grafted into the inter-scapular region or into the renal capsule of immunodeficient or severe-combined immuno-deficiency mice. When the tumour volume reaches the ethical limit, the tumour is removed and fragmented as follows: a part is grafted to a new set of mice; a second part is fixed in formalin and embedded in paraffin for histological studies; a third part is frozen in liquid nitrogen for molecular studies; and a last part is frozen in a 10% DMSO solution to generate a viable tissue stock. These steps are repeated for the F1, F2 and F3 generation. A xenograft will be considered as well-established in the third generation (F3).

The patient-derived tumour xenograft platform called XenTech will generate the PDX. All PDX will be established as approved by the ethical authorisation #16569: « Développement d’une collection de modèles de tumeurs humaines greffés sur souris (PDX) ». The different steps of the development of an established xenograft model are summarized in Figure 2. Overall, fresh surgical tissue will be cut into small fragments and grafted into the inter-scapular region or into the renal capsule of 6- to 13-week-old female immunodeficient or severe-combined immuno-deficiency (SCID) or non-obese diabetic SCID mice. Although the use of extracellular matrix tends to increase xenograft take rates, tumor fragments will be implanted without any matrix. Mice should weigh 18g at 6 months. At the onset of tumour growth, a latency period of 1 to 9 months is expected. The mouse generation with the patient derived graft will be called F0 and the following generations will be numbered F1, F2, and F3. When the tumour volume reaches the ethical limit (10% of the total weight of the mouse), the tumour is removed and fragmented as follows: a part is grafted to a new set of mice; a second part is fixed in formalin and embedded in paraffin for histological studies; a third part is frozen in liquid nitrogen for molecular studies; and a last part is frozen in a 10% DMSO solution to generate a viable tissue stock. These steps are repeated for the F1, F2 and F3 generation. A xenograft will be considered as well-established in the third generation (F3). When it is necessary, the animal will be sacrificed by cervical dislocation. For the duration of the study, mice will be housed, by a maximum of 6, in individually ventilated cages. They will be housed in a light-dark cycle with temperature and hygrometry control. The mice will have throughout the duration of the project a complete diet and drinking water. For the ER+ PDX models, mice will receive 17β-estradiol supplementation in the drinking water, useful for the establishment of these models ^10^.

### Sample selection

Inclusion and exclusion criteria are presented in Table 1. Briefly, adult women (18 years or older) with triple-negative or luminal B breast cancer in neoadjuvant, adjuvant or metastatic setting will be included.

**Table 1.** Inclusion and exclusion criteria.

Inclusion criteria	• Female • Age ≥ 18 years • ECOG (Eastern Cooperative Oncology Group) performance status ≤ 2 • Women with : ○ high grade metaplastic triple-negative (TN) breast cancer, histologically proven before treatment, receiving neoadjuvant chemotherapy and having, after treatment, a breast residue of at least 15 mm on the specimen. The mammary residue will measure at least 15 mm on the mammography performed at the end of neoadjuvant treatment OR ○ high grade metaplastic triple-negative (TN) breast cancer, histologically proven before treatment, treated by primary surgery with a tumour size of at least 15 mm on the specimen OR ○ inflammatory TN breast cancer (T4d), histologically proven prior to treatment, receiving neoadjuvant chemotherapy and having, after treatment, a breast residue of at least 15 mm on the specimen. The mammary residue will measure at least 15 mm on the mammography performed at the end of the neoadjuvant treatment OR ○ TN breast cancer other than non-metaplastic or inflammatory, histologically proven prior to treatment, receiving neoadjuvant chemotherapy and having, after treatment, a mammary residue of at least 30 mm on the specimen. The mammary residue will measure at least 15 mm on the mammography performed at the end of the neoadjuvant treatment OR ○ Luminal B breast cancer, histologically proven prior to treatment, receiving neoadjuvant chemotherapy and having, after treatment, a mammary residue of at least 30 mm on the specimen. The mammary residue will measure at least 15 mm on the mammography performed at the end of the neoadjuvant treatment OR ○ Metastatic TN or luminal B breast cancer, histologically proven at diagnosis, with an operable metastasis and having after chemotherapy a residue of at least 10 mm on the surgical specimen. Residual metastasis will measure at least 15 mm on imaging. • Patients in a metastatic situation can be included regardless of the therapeutic line • Affiliation to social security • Signature of the participation consent of the study
Exclusion criteria	• Pregnant woman • Patient deprived of liberty by court or administration decision • In neoadjuvant situation: neoadjuvant treatment by radiotherapy or hormone therapy • Refusal to participate to the study

### Recruitment and consent

Eligible patients will be offered the opportunity to participate in the study by their oncologist or their surgeon. Patients who agree to participate in this study will provide written informed consents (clinical consent and genetic consent) for enrolment.

### Sample size calculation

This feasibility study aims to obtain xenografts from tumours that are either rare or tumours that are resistant to treatment and therefore difficult to establish. We consider that a minimum of five successful grafts would meet this objective. Under these conditions, knowing the graft success rate specific to each histological type and the proportion of luminal B (2/3) and triple-negative breast cancers (1/3), we calculate the number of subjects required so that the lower bound of the 95% confidence interval (CI) of the success rate multiplied by the number of subjects is ≥5.

To obtain a xenograft, the average rate is 30% from triple-negative tumours and 10% from luminal B tumours. Taking these data into account, we will include 2/3 luminal B tumours and 1/3 triple negative tumours: the expected rate of successful xenografts will be approximately 17%. The number of patients needed to be included is therefore at least 65: lower bound of the CI-95% of 17% for 65 subjects = 8% or 5 patients (8%×65=5.2).

Since for some patients the tumour will be of sufficient size for imaging, but of insufficient size in the operating room, we consider that this number should be increased by 30%, to a total number of 85 patients.

### Study objectives and data collections

The primary objective of the XENOBREAST trial is to establish xenografts from tumour samples of patients with triple negative or luminal B breast cancer in neoadjuvant, adjuvant or metastatic setting. Furthermore, the study aims to investigate histomolecular characteristics of the established PDX and to compare these characteristics with the initial histomolecular profile of the collected tumours.

Data collected are the patient's age (month and year of birth), pathology, treatments received, response to treatments, date and nature of surgery (primary tumour, metastasis), data concerning exomes (constitutional and tumour), as well as histomolecular profiles of the initial tumour and of the different xenograft generations. To define these histomolecular profiles we will quantify the expression of the oestrogen, progesterone and androgen receptors by immunohistochemistry, the amplification status of the *ERBB2* gene, and the fraction of tumour cells expressing Ki67. Finally, tumours will be classified into molecular classes according to the above-mentioned data: luminal A, luminal B, HER2-enriched or triple negative.

Data collected and transmitted to the sponsor of the study by the investigators will be pseudonymized. Study data will not contain any names or other personal identifiers such as addresses. Patients included in the trial will be identified by a code specific to this trial. The investigator will have access to the correspondence table between the patient's last name, first name, date of birth and the code assigned in the trial.

### Statistical analysis

***Primary analysis.*** The main outcome of this feasibility trial is the number of successful xenografts obtained, a xenograft being considered successful when it reaches the F3 generation and its molecular subtype defined by immunohistochemistry has remained identical to the original tumour. The percentage of tumours yielding a successful xenograft will also be calculated, along with a 95% confidence interval. The feasibility will be considered acceptable if at least five successful transplants are obtained.

***Secondary analysis.*** A diversity analysis of genomic data from the tumour exomes and the constitutional exome will be performed. Differential analyses using bioinformatics tools adapted to these data could be envisaged if the number of patients allows it. We will describe in detail the collected tumours (description of the population, histomolecular profile, anatomopathological data, etc.). Qualitative characteristics will be described using their number and frequency, and quantitative characteristics (age at diagnosis, tumour size, etc.) using standard distribution parameters: mean, median, standard deviation, extremes, normality.

The characteristics of the tumours will also be described according to whether or not a xenograft was successful. These characteristics will also be compared, if the sample sizes allow it, using Fisher’s exact test, and Welch's t-test or the non-parametric Mann-Whitney U-test if needed. All tests will be two-sided and the statistical significance threshold will be generally set at 0.05 except in case of differential analyses on the exome data where multiple testing corrections will be applied.

### Ethical considerations

The XENOBREAST trial has been approved by an ethics committee (Sud Méditerranée IV – Montpellier) on April 2020 (Reference: 20 03 02 and ID-RCB number: 2020-A00398-31). It is conducted notably in accordance with the Declaration of Helsinki and General Data Protection Regulation (GDPR).

Study data and finding will be published in peer-reviewed medical journals. We plan to present the study and all data at national congresses and conferences.

### Trial status

Participant recruitment is expected to begin in October 2020 and to finish in October 2023. The approved protocol is version 02, 24/03/2020.

## Conclusion

The XENOBREAST study a feasibility pilot trial that will allow us to estimate the success rate of xenografts and to estimate PDX drift by comparing the histomolecular profile of the PDX to that of the tumour. In perspective, the generation of PDX from rare and chemo-resistant tumours would allow for testing new treatments before their administration *in vivo*. In the long term, the establishment of PDX from primary mammary tumours or after neoadjuvant treatment would allow a better understanding of the therapeutic response. Moreover, it could be a great model to explore tumour evolution patterns during metastatic progression and to observe tumour resistance mechanisms in non-metastatic tumours.

## Data availability

No data are associated with this article.
