# Supplementary material for: XENOBREAST Trial: A prospective study of xenografts establishment from surgical specimens of patients with triple negative or luminal b breast cancer
Source: F1000Res. 2021 Jun 21;9:1219. Originally published 2020 Oct 9. [Version 3] doi: 10.12688/f1000research.26873.3 (PMC8258709; doi:10.12688/f1000research.26873.3)
Supplement: Peer review history

# Peer review: Reviewer response for version 3

**DOI:** 10.5256/f1000research.57809.r87975

**Published:** 2021-07-05

**Authors:** Fares Al-Ejeh

**Affiliations:** 1 QIMR Berghofer Medical Research Institute, Royal Brisbane Hospital, Herston, Queensland, 4029, Australia; 2 Qatar Biomedical Research Institute, Doha, Qatar

The authors' revisions address the points raised on the previous version.

Is the study design appropriate for the research question?

Yes

Is the rationale for, and objectives of, the study clearly described?

Yes

Are sufficient details of the methods provided to allow replication by others?

Partly

Are the datasets clearly presented in a useable and accessible format?

Not applicable

Reviewer Expertise:

Breast cancer research and translational in vitro and in vivo models, including PDXs and PDCLs. My area of research is cancer signalling and identification of biomarkers and therapeutic targets from clinical samples to investigate their role and clinical potential in preclinical models.

I confirm that I have read this submission and believe that I have an appropriate level of expertise to confirm that it is of an acceptable scientific standard.

# Peer review: Reviewer response for version 2

**DOI:** 10.5256/f1000research.54662.r84566

**Published:** 2021-05-28

**Authors:** Fares Al-Ejeh

**Affiliations:** 1 QIMR Berghofer Medical Research Institute, Royal Brisbane Hospital, Herston, Queensland, 4029, Australia; 2 Qatar Biomedical Research Institute, Doha, Qatar

In this protocol, the investigators aim to establish PDXs from the more aggressive subtypes of breast cancer, TNBC and Luminal B for future use in translational research.

 Overall, the protocol for the XENOBREAST study is clearly described.  However, some edits and inclusion of suggested literature below should be included.

- The abstract and the introduction makes a statement that " *Most of the available PDX, derived from breast cancer, were generated from metastatic tumours.*" - This is not accurate. Indeed, most of the commonly breast cancer cells lines established few decades ago were from metastases (mainly pleural effusion).  In contrast, PDXs and organoids, which have been xenotransplated into mice and recapitulate the primary tumors,  are more commonly derived from primary breast cancer tissue (Bruna *et al.* (2016 ^1^), DeRose *et al. *(2011 ^2^), Sachs *et al.* (2018 ^3^), Rosenbluth *et al. *(2020 ^4^)). Also see the following link. This statement needs to be corrected and to cite this relevant literature.
- The involvement of the company XenTech, who already have established breast cancer xenografts, needs to be clarified. - Is the company only providing a service or will maintain, use and sell those established xenografts?  If commercial rights are involved in favor of XenTech, it is important to state that patients will be made aware during consent.
- The choice of into the inter-scapular region or into the renal capsule as the injection sites are unusual (not orthotopic) for breast cancer. - PDXs have been shown to undergo mouse-specific tumor evolution (Ben-David *et al*. (2017 ^5^)), which should be noted and cited in this manuscript. The environment where PDXs are established will also have an impact on the molecular profile of the tumor, thus the choice to avoid the mammary gland to establish PDXs in this study needs to be clearly explained and justified.
- Moreover, it is not clear if estrogen pellets (not in the feed) will be implanted in the mice for the establishment and maintenance of luminal B PDXs.
- Considering the above, why is not the design considering cryopreservation of tumor pieces to store alongside the injection of some pieces into mice?  This will allow the option to implant the PDXs in the mammary glands if the investigators find that the inter-scapular region and the renal capsule sites cause genetic/molecular drift in the established PDXs compared to the original tissue and during PDX passage (F0-F3).

Is the study design appropriate for the research question?

Yes

Is the rationale for, and objectives of, the study clearly described?

Yes

Are sufficient details of the methods provided to allow replication by others?

Partly

Are the datasets clearly presented in a useable and accessible format?

Not applicable

Reviewer Expertise:

Breast cancer research and translational in vitro and in vivo models, including PDXs and PDCLs. My area of research is cancer signalling and identification of biomarkers and therapeutic targets from clinical samples to investigate their role and clinical potential in preclinical models.

I confirm that I have read this submission and believe that I have an appropriate level of expertise to confirm that it is of an acceptable scientific standard, however I have significant reservations, as outlined above.

## References

[1] : A Biobank of Breast Cancer Explants with Preserved Intra-tumor Heterogeneity to Screen Anticancer Compounds. *Cell* .2016;167(1) : 10.1016/j.cell.2016.08.041 260-274.e22 10.1016/j.cell.2016.08.041 PMC503731927641504

[2] : Tumor grafts derived from women with breast cancer authentically reflect tumor pathology, growth, metastasis and disease outcomes. *Nat Med* .2011;17(11) : 10.1038/nm.2454 1514-20 10.1038/nm.2454 22019887PMC3553601

[3] : A Living Biobank of Breast Cancer Organoids Captures Disease Heterogeneity. *Cell* .2018;172(1-2) : 10.1016/j.cell.2017.11.010 373-386.e10 10.1016/j.cell.2017.11.010 29224780

[4] : Organoid cultures from normal and cancer-prone human breast tissues preserve complex epithelial lineages. *Nature Communications* .2020;11(1) : 10.1038/s41467-020-15548-7 10.1038/s41467-020-15548-7 PMC713620332249764

[5] : Patient-derived xenografts undergo mouse-specific tumor evolution. *Nat Genet* .2017;49(11) : 10.1038/ng.3967 1567-1575 10.1038/ng.3967 28991255PMC5659952

# Response

**Published:** 2021-06-14

**Authors:** Hugo Veyssière

**Affiliations:** Centre Jean Perrin, France

Thank you for your report and comments.

 The abstract and the introduction make a statement that "Most of the available PDX, derived from breast cancer, were generated from metastatic tumours." - This is not accurate. Indeed, most of the commonly breast cancer cells lines established few decades ago were from metastases (mainly pleural effusion).  In contrast, PDXs and organoids, which have been xenotransplanted into mice and recapitulate the primary tumors,  are more commonly derived from primary breast cancer tissue (Bruna et al. (2016), DeRose et al. (2011), Sachs et al. (2018), Rosenbluth et al. (2020)). Also see the following link. This statement needs to be corrected and to cite this relevant literature.

**Response: The statement has been modified and relevant literature is cited in the introduction.**

  The involvement of the company XenTech, who already have established breast cancer xenografts, needs to be clarified. - Is the company only providing a service or will maintain, use and sell those established xenografts?  If commercial rights are involved in favor of XenTech, it is important to state that patients will be made aware during consent.

**Response:** **XenTech may commercialize the product of its research (this sentence is present in the consent). And patients will be made aware during consent.**

**We added a sentence in the “Recruitment and consent” paragraph:** **“** **XenTech may commercialize the product of its research, and patients will be made aware during consent.”**

****

 The choice of into the inter-scapular region or into the renal capsule as the injection sites are unusual (not orthotopic) for breast cancer. - PDXs have been shown to undergo mouse-specific tumor evolution (Ben-David et al. (20175)), which should be noted and cited in this manuscript. The environment where PDXs are established will also have an impact on the molecular profile of the tumor, thus the choice to avoid the mammary gland to establish PDXs in this study needs to be clearly explained and justified.

**Response:** **For breast cancer PDXs, the placement in the interscapular region is a choice made by Xentech when it began generating breast cancer PDXs. This implant site allows easy access to a well vascularized region with tissue that in female mice is in continuity with the mammary gland. We added a sentence in the “PDX generation” paragraph.**

 Moreover, it is not clear if estrogen pellets (not in the feed) will be implanted in the mice for the establishment and maintenance of luminal B PDXs.

**Response: As mentioned in the manuscript,** **for the ER+ PDX models, mice will receive 17β-estradiol supplementation in the drinking water, useful for the establishment and the maintenance of these models**

 Considering the above, why is not the design considering cryopreservation of tumor pieces to store alongside the injection of some pieces into mice?  This will allow the option to implant the PDXs in the mammary glands if the investigators find that the inter-scapular region and the renal capsule sites cause genetic/molecular drift in the established PDXs compared to the original tissue and during PDX passage (F0-F3).

**Response:** **As we wrote in the manuscript, “a part is grafted to a new set of mice; a second part is fixed in formalin and embedded in paraffin for histological studies; a third part is frozen in liquid nitrogen for molecular studies, and a last part is frozen in a 10% DMSO solution to generate a viable tissue stock.”**

**Thus, we do want to have a viable tissue stock. However, because the surgical specimen will be limited we are not sure that we will have enough material to cryopreserve a part of the tumors.**

# Peer review: Reviewer response for version 2

**DOI:** 10.5256/f1000research.54662.r81197

**Published:** 2021-03-12

**Authors:** Jessica Finlay-Schultz

**Affiliations:** 1 Department of Pathology, University of Colorado Anschutz Medical Campus, Aurora, Colorado, 80309, USA

I have no further comments.

Is the study design appropriate for the research question?

Yes

Is the rationale for, and objectives of, the study clearly described?

Yes

Are sufficient details of the methods provided to allow replication by others?

No

Are the datasets clearly presented in a useable and accessible format?

Not applicable

Reviewer Expertise:

My expertise is in the establishment and propagation of breast cancer PDX models, primarily from hormone receptor positive tumors. My area of research includes estrogen and progesterone receptor signaling, crosstalk, and transcriptional regulation in breast cancer models, including PDX and cell lines derived from primary tissue and PDX models.

I confirm that I have read this submission and believe that I have an appropriate level of expertise to confirm that it is of an acceptable scientific standard.

# Peer review: Reviewer response for version 1

**DOI:** 10.5256/f1000research.29677.r76613

**Published:** 2021-01-26

**Authors:** Jessica Finlay-Schultz

**Affiliations:** 1 Department of Pathology, University of Colorado Anschutz Medical Campus, Aurora, Colorado, 80309, USA

In this manuscript, Veyssière *et al.* outline a study protocol for generation of PDX models from patient tumors of the triple negative or luminal B molecular subtypes. The overall study metrics and patient enrollment numbers appear sound, though the methods are light on experimental details. The writing is clear and concise, though the phrase “primitive mammary tumours” should be replaced with “primary mammary tumours” in the Abstract. While I understand that the authors will be using a private company to generate these PDX models, some details of their generation should be included, and the group would benefit from reviewing additional established PDX generation protocols.

1. Kabos *et al. *(PMID 22821401) ^1^ have established that supplementation with 17β-estradiol is useful for the establishment of ER+ PDX models. Is there a plan for hormone supplementation for the Luminal B portion of this study?
2. In addition, breast cancer PDX models are often placed into the #4 mammary fat pad of these animals. Is there a specific reason behind avoiding this placement in this study?
3. Will there be the use of an extracellular matrix for implantation of the primary tumor? While ECM use tends to increase xenograft take rates, there have been historical links between some ECM producers and the propagation of LDEV (Lactate Dehydrogenase Elevating Virus) with tumor tissues.
4. Review and citation of Dobrolecki et al (PMID 28025748) ^2^ will help answer some major questions in the breast tumor PDX field, and this should be referenced in your study, as it is a comprehensive review of the majority of well-established breast tumor PDX banks.
5. Please include that the primary molecule that differs between Luminal A and Luminal B tumors is Ki67. While Luminal B tumors may often exhibit lower HR expression, it is not the main factor used to classify these tumors.

Is the study design appropriate for the research question?

Yes

Is the rationale for, and objectives of, the study clearly described?

Yes

Are sufficient details of the methods provided to allow replication by others?

No

Are the datasets clearly presented in a useable and accessible format?

Not applicable

Reviewer Expertise:

My expertise is in the establishment and propagation of breast cancer PDX models, primarily from hormone receptor positive tumors. My area of research includes estrogen and progesterone receptor signaling, crosstalk, and transcriptional regulation in breast cancer models, including PDX and cell lines derived from primary tissue and PDX models.

I confirm that I have read this submission and believe that I have an appropriate level of expertise to confirm that it is of an acceptable scientific standard, however I have significant reservations, as outlined above.

## References

[1] : Patient-derived luminal breast cancer xenografts retain hormone receptor heterogeneity and help define unique estrogen-dependent gene signatures. *Breast Cancer Res Treat* .2012;135(2) : 10.1007/s10549-012-2164-8 415-32 10.1007/s10549-012-2164-8 22821401PMC3818141

[2] : Patient-derived xenograft (PDX) models in basic and translational breast cancer research. *Cancer Metastasis Rev* .35(4) : 10.1007/s10555-016-9653-x 547-573 10.1007/s10555-016-9653-x 28025748PMC5396460

# Response

**Published:** 2021-02-11

**Authors:** Hugo Veyssière

**Affiliations:** Centre Jean Perrin, France

Thank you for taking the time to review our manuscript. We appreciate your helpful comments.

 1) Kabos *et al. *(PMID 22821401) ^1^ have established that supplementation with 17β-estradiol is useful for the establishment of ER+ PDX models. Is there a plan for hormone supplementation for the Luminal B portion of this study?

**All the ER+ PDX models are established in mice receiving 17β-estradiol supplementation in the drinking water. A sentence has been added in our manuscript.**

 2) In addition, breast cancer PDX models are often placed into the #4 mammary fat pad of these animals. Is there a specific reason behind avoiding this placement in this study?

**For breast cancer PDXs, the placement in the interscapular region is a choice made by Xentech when it began generating breast cancer PDXs. This implant site allows easy access to a well vascularized region with tissue that in female mice is in continuity with the mammary gland.**

 3) Will there be the use of an extracellular matrix for implantation of the primary tumor? While ECM use tends to increase xenograft take rates, there have been historical links between some ECM producers and the propagation of LDEV (Lactate Dehydrogenase Elevating Virus) with tumor tissues. 

**We do not foresee the use of any matrix during the first implantation, only tumor fragments will be implanted.**

 4) Review and citation of Dobrolecki et al (PMID 28025748) ^2^ will help answer some major questions in the breast tumor PDX field, and this should be referenced in your study, as it is a comprehensive review of the majority of well-established breast tumor PDX banks.

**Dobrolecki et al. has been added to references.**

 5) Please include that the primary molecule that differs between Luminal A and Luminal B tumors is Ki67. While Luminal B tumors may often exhibit lower HR expression, it is not the main factor used to classify these tumors.

**Precisions have been added in the article.**
